# Five-Aminolevulinic Acid (5-ALA) Induces Heme Oxygenase-1 and Ameliorates Palmitic Acid-Induced Endoplasmic Reticulum Stress in Renal Tubules

**DOI:** 10.3390/ijms241210151

**Published:** 2023-06-15

**Authors:** Shintaro Hamada, Yukari Mae, Tomoaki Takata, Hinako Hanada, Misaki Kubo, Sosuke Taniguchi, Takuji Iyama, Takaaki Sugihara, Hajime Isomoto

**Affiliations:** Division of Gastroenterology and Nephrology, Faculty of Medicine, Tottori University, Tottori 683-8504, Japan

**Keywords:** Nrf2, Bach1, steatosis, kidney, lipotoxicity

## Abstract

Steatosis, or ectopic lipid deposition, is the fundamental pathophysiology of non-alcoholic steatohepatitis and chronic kidney disease. Steatosis in the renal tubule causes endoplasmic reticulum (ER) stress, leading to kidney injury. Thus, ER stress could be a therapeutic target in steatonephropathy. Five-aminolevulinic acid (5-ALA) is a natural product that induces heme oxygenase (HO)-1, which acts as an antioxidant. This study aimed to investigate the therapeutic potential of 5-ALA in lipotoxicity-induced ER stress in human primary renal proximal tubule epithelial cells. Cells were stimulated with palmitic acid (PA) to induce ER stress. Cellular apoptotic signals and expression of genes involved in the ER stress cascade and heme biosynthesis pathway were analyzed. The expression of glucose-regulated protein 78 (GRP78), a master regulator of ER stress, increased significantly, followed by increased cellular apoptosis. Administration of 5-ALA induced a remarkable increase in HO-1 expression, thus ameliorating PA-induced GRP78 expression and apoptotic signals. BTB and CNC homology 1 (*BACH1*), a transcriptional repressor of HO-1, was significantly downregulated by 5-ALA treatment. HO-1 induction attenuates PA-induced renal tubular injury by suppressing ER stress. This study demonstrates the therapeutic potential of 5-ALA against lipotoxicity through redox pathway.

## 1. Introduction

Metabolic syndrome, which includes obesity, hyperglycemia, hypertension, and dyslipidemia, is a major public health problem strongly associated with the progression of cardiovascular disease and chronic kidney disease (CKD) [1,2,3,4]. Diabetic nephropathy and nephrosclerosis are the two major etiologies of end-stage renal disease in Japan [5]. The importance of controlling glucose levels and blood pressure is widely recognized. In addition to conventional hypoglycemic agents and insulin, newly developed agents such as glucagon-like peptide-1 and its related peptide, dipeptidyl peptide IV, sodium-glucose cotransporter-2 (SGLT2) inhibitors, and imeglimin have improved the therapeutic strategies for the management of blood glucose levels in patients with diabetes mellitus [6,7]. Similarly, recent advances in antihypertensive agents including mineral corticoid receptor blockade and angiotensin receptor-neprilysin inhibitor have made the management of blood pressure easier to achieve its target levels. Although there are currently multiple therapeutic approaches for glucose metabolism and hypertension [8,9], the therapeutic agents for dyslipidemia are still limited. Dyslipidemia aggravates CKD either by itself or in combination with impaired glucose metabolism. Dyslipidemia is often accompanied by hepatic insults. Non-alcoholic fatty liver disease (NAFLD) and non-alcoholic steatohepatitis (NASH) are emerging chronic liver diseases associated with dyslipidemia, which develops without excess alcohol consumption. Recently, because of the similarity in the risk factors and epidemiological evidence that patients with metabolic syndrome often present both CKD and NASH, a potential association has been suggested between the underlying pathophysiology of CKD and NASH [10]. In the liver, the multiple hits hypothesis has been proposed as the underlying pathogenesis of NASH. Endoplasmic reticulum (ER) stress, induced by steatosis or ectopic lipid deposition in hepatocytes, is one such mechanism associated with NASH pathophysiology [11,12]. Similar to the liver, steatosis in renal tubules is closely associated with kidney injury via the induction of ER stress [13,14]. Therefore, ER stress could be a therapeutic target to protect the kidneys against lipotoxicity.

Five-aminolevulinic acid (5-ALA) is a natural amino acid essential for the heme biosynthesis pathway. The metabolism of 5-ALA and heme biosynthesis are rate-limiting, mediated by eight consecutive enzymes and three transporters. ALA is synthesized endogenously from glycine and succinyl CoA by 5-ALA synthase under normal conditions. Exogenous ALA is absorbed through oligopeptide transporter-1, expressed in the cell membrane surface, then metabolized to coproporphyrinogen III, subsequently transported into mitochondria through ATP-binding cassette transporter B6. In the mitochondria, coproporphyrinogen III is then metabolized to protoporphyrin IX (PpIX). PpIX is eliminated from the cell through the ATP-binding cassette transporter G2 or metabolized to heme within the mitochondria by a rate-limiting enzyme, ferrochelatase [15]. The heme biosynthesis pathway has received considerable attention for cancer diagnosis and therapy [16]. Cancer cells show altered enzymatic activity to catabolize PpIX, causing the accumulation of metabolites within the cells [17]. Based on the characteristics of PpIX, which emits red fluorescence upon irradiation with blue light, 5-ALA has been used for the photodynamic diagnosis of cancers [15]. In addition to its role in heme biosynthesis, 5-ALA induces the expression of heme oxygenase (HO)-1, a heme-degrading enzyme [18]. HO-1 plays an essential role in degrading heme to iron, carbon monoxide, and bilirubin, and it attenuates inflammation and apoptosis [19]. HO-1 induction prevents endothelial cell death by attenuating ER stress [20]. Therefore, we hypothesized that 5-ALA protects the renal tubules against lipotoxicity. In this study, we investigated the therapeutic potential of 5-ALA against steatosis-induced ER stress and apoptosis of renal tubular cells. 

## 2. Results

### 2.1. PA Induced Cellular Apoptosis in RPTECs

**Figure 1:**
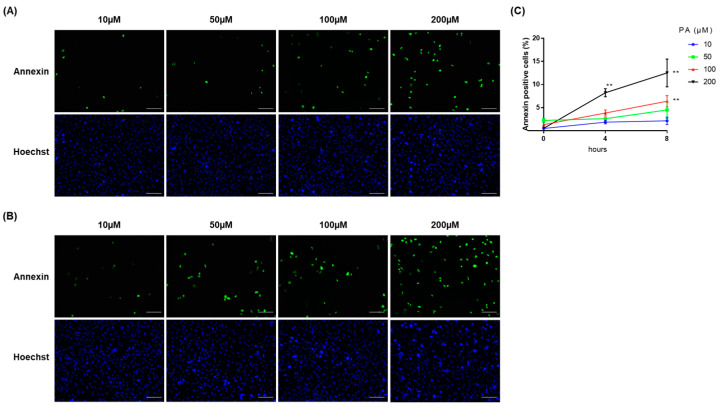
PA-induced apoptosis in renal tubular cells. Palmitic acid (PA)-induced cellular apoptosis in immortalized human primary renal proximal tubule epithelial cells (RPTECs) was evaluated via Annexin V/SYTOX staining. Representative fluorescent images for Annexin V/SYTOX staining and Hoechst33342 in RPTECs after administration of PA for (**A**) 4 h and (**B**) 8 h. Green indicates Annexin positive cells. Blue indicates Hoechst positive cells. The RPTECs were treated with PA at the concentrations of 10, 50, 100, and 200 μM. Bars indicate 100 μm. (**C**) Quantification of Annexin staining was expressed as the ratio of Annexin-positive cells to the Hoechst33342-positive cells. The quantification is based on at least four randomly captured fields in each condition. Bars indicate means ± SEM; ** *p* < 0.01 compared to baseline (one-way analysis of variance with Tukey’s post hoc test).

**Figure 2:**
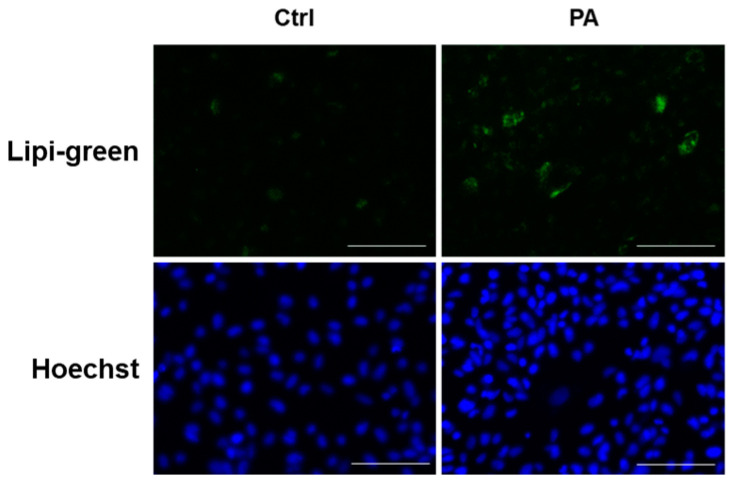
PA-induced steatosis in renal tubular cells. Representative fluorescent images for Lipi-green and Hoechst33342 staining in RPTECs treated with PA for 4 h. Green indicates Lipigreen positive cells, whereas blue indicates Hoechst positive cells. Bars indicate 100 μm.

We first investigated the induction of ER stress by excess fatty acid in renal tubular cells. Palmitic acid (PA) is shown to induce steatosis and ER stress in hepatocytes [21,22] and PA-induced lipid accumulation in proximal tubular cell has been demonstrated [23]; therefore, PA was used for fatty acid stimulation to investigate lipotoxicity in immortalized human primary renal proximal tubule epithelial cells (RPTECs). The cells were incubated with bovine serum albumin (BSA) or PA-complexed BSA at concentrations of 10, 50, 100, and 200 μM of PA, and cellular apoptosis was investigated. We observed that the ratio of apoptotic cells significantly increased, in a time- and dose-dependent manner, at 100 μM of PA for 8 h and 200 μM of PA for 4 h and 8 h (Figure 1). No significant increase was observed at 10 and 50 μM of PA treatment. As treatment with PA at 200 μM for 4 h is enough to induce renal tubular cell injury, the cells were treated with this condition in the following experiments. We further confirmed lipid accumulation in RPTECs (Figure 2).

### 2.2. Treatment with 5-ALA Attenuated ER Stress and Induced HO-1 Expression

**Figure 3:**
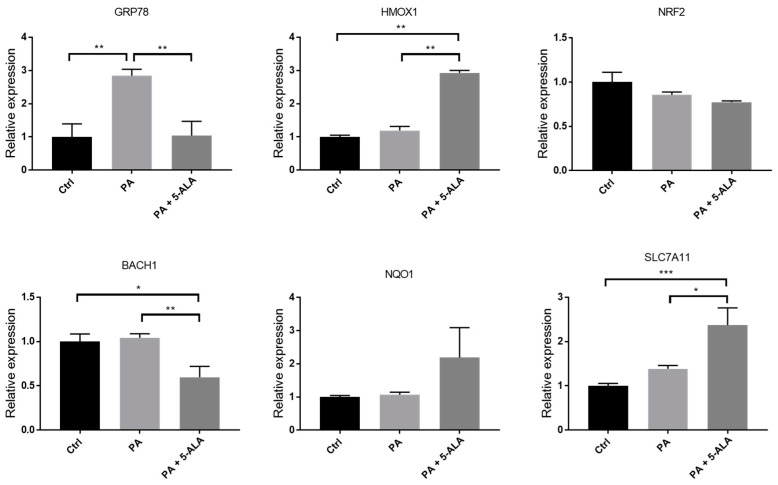
Changes in the expression of heme biosynthesis and ER stress-associated genes after PA and 5-ALA. The mRNA expression of genes, including *GRP78, HMOX1, NRF2, BACH1*, *NQO1*, and *SLC7A11***,** after administration of PA and 5-ALA were measured via real-time RT-PCR. The cells were treated with 1% bovine serum albumin (Ctrl), 200 μM PA, or PA with 200 μM 5-ALA for 4 h. The results are expressed as relative levels to control. The quantification is based on at least five repetitive experiments. Bars indicate means ± SEM; * *p* < 0.05; ** *p* < 0.01; *** *p* < 0.001 compared to baseline (one-way analysis of variance with Tukey’s post hoc test). PA, palmitic acid; 5-ALA, 5-aminolevulinic acid.

**Figure 4:**
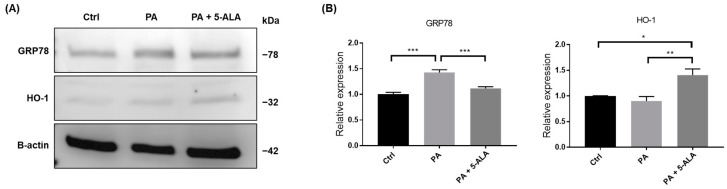
Changes in the expression of heme biosynthesis and ER stress-associated proteins after PA and 5-ALA. (**A**) Representative images of the blots for GRP78, HO-1, and β-actin after administration of PA and 5-ALA. (**B**) Quantification of the signal intensities expressed relative to control. The cells were treated with 1% bovine serum albumin (Ctrl), 200 μM PA, or PA with 200 μM 5-ALA for 4 h. The quantification is based on at least three repetitive experiments. Bars indicate means ± SEM; * *p* < 0.05; ** *p* < 0.01; *** *p* < 0.001 compared to baseline (one-way analysis of variance with Tukey’s post hoc test). PA, palmitic acid; 5-ALA, 5-aminolevulinic acid.

The expression of genes involved in heme biosynthesis cascade and ER stress pathway was measured using real-time reverse transcription (RT)-PCR. PA induced the expression of glucose-regulated protein 78 (*GRP78*), the master regulator of the ER stress response in PRTECs. Administration of 5-ALA significantly suppressed PA-induced *GRP78* expression. The expression of *HMOX1*, which encodes HO-1, was not changed by PA alone; however, it markedly increased following 5-ALA administration. Although the levels of nuclear factor erythroid 2-related factor 2 (*NRF2*), the upstream master regulator of cellular redox homeostasis, did not change after the administration of 5-ALA, those of BTB and CNC homology 1 (*BACH1*), a transcriptional repressor of *HMOX1*, were significantly decreased. The expression in *NQO1*, a target gene of *NRF2* and *BACH1* playing an antioxidant role, showed an increasing trend after 5-ALA treatment, but the change was not significant. The expression of *SLC7A11*, Nrf2-regulated gene responsible for cysteine and glutamate transport, significantly increased after 5-ALA treatment (Figure 3). We also investigated the changes in proteins associated with ER stress and the heme biosynthesis pathway. As a result, 5-ALA significantly induced HO-1 and ameliorated the PA-induced ER stress (Figure 4).

### 2.3. Treatment with 5-ALA Attenuated PA-Induced Caspase-3/7 Activity and Apoptosis

**Figure 5:**
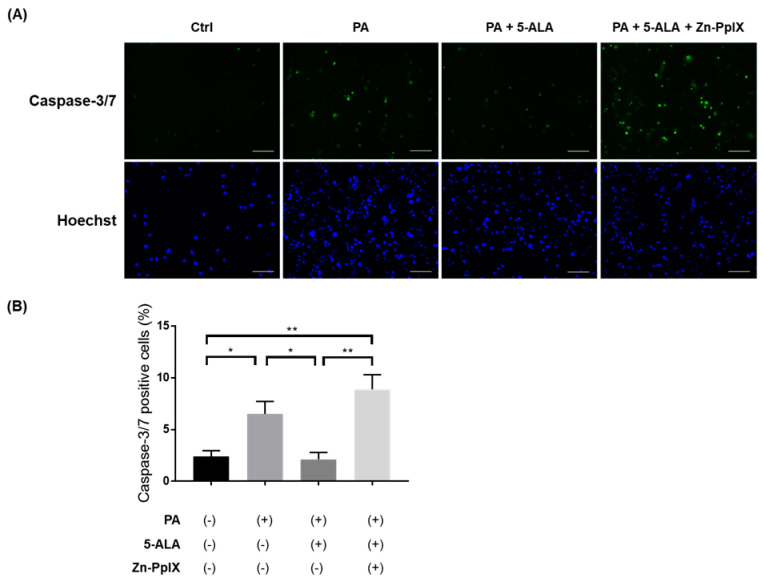
Caspase assay in RPTECs. (**A**) Representative fluorescent images for caspase-3/7 staining and Hoechst33342 in RPTECs after administration of PA, 5-ALA, and Zn-PpIX. The cells were treated with 1% BSA (Ctrl) or 200 μM PA for 4 h. The concentrations of 5-ALA and Zn-PpIX were at 200 μM and 10 μM, respectively. Green indicates caspase-3/7 positive cells, whereas blue indicates Hoechst positive cells. Bars indicate 100 μm. (**B**) Quantification of caspase-3/7 staining showing the percentage of caspase-3/7-positive cells in each condition. The quantification is based on at least five randomly captured fields. Bars indicate means ± SEM; * *p* < 0.05, ** *p* < 0.01 (one-way analysis of variance with Tukey’s post hoc test). RPTEC, immortalized human primary renal proximal tubule epithelial cell; PA, palmitic acid; 5-ALA, 5-aminolevulinic acid; Zn-PpIX, zinc-protoporphyrin IX; BSA, bovine serum albumin.

**Figure 6:**
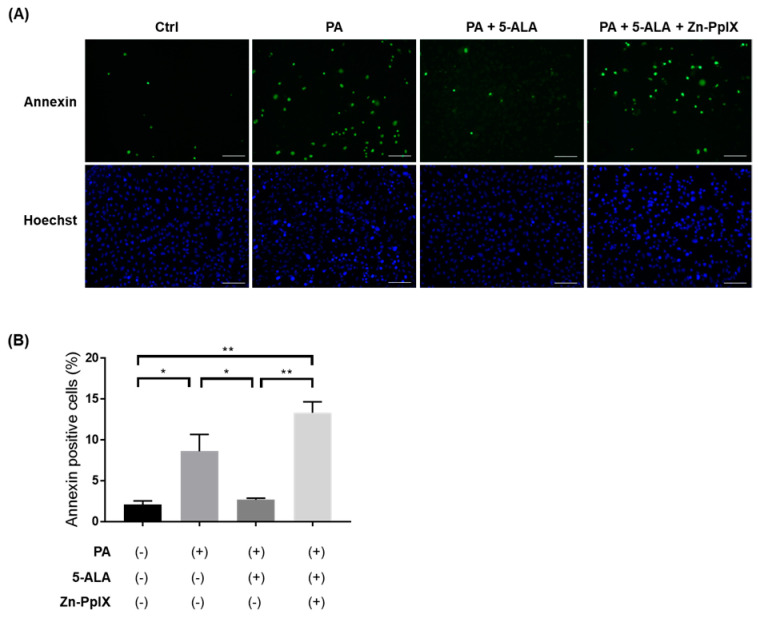
Cellular apoptosis assay in RPTECs. (**A**) Representative fluorescent images for Annexin V/SYTOX staining and Hoechst33342 in RPTECs after administration of 1% BSA, PA, 5-ALA, and Zn-PpIX for 4 h. The concentrations of 5-ALA and Zn-PpIX were at 200 μM and 10 μM, respectively. Green indicates annexin positive cells. Blue indicates Hoechst positive cells. Bars indicate 100 μm. (**B**) Quantification of Annexin V/SYTOX staining showing the percentage of apoptotic cells. The quantification is based on at least four randomly captured fields. Bars indicate means ± SEM; * *p* < 0.05, ** *p* < 0.01 (one-way analysis of variance with Tukey’s post hoc test). RPTEC, immortalized human primary renal proximal tubule epithelial cell; PA, palmitic acid; 5-ALA, 5-aminolevulinic acid; Zn-PpIX, zinc-protoporphyrin IX; BSA, bovine serum albumin.

To investigate the effects of 5-ALA on lipotoxicity and apoptosis in renal tubules, we evaluated the activity of caspase-3/7, which directly mediates apoptosis. The proportion of active caspase-3/7 cell number significantly increased under PA administration, and 5-ALA attenuated their activity. Furthermore, Zn-PpIX, a HO-1 inhibitor, reversed the protective effects of 5-ALA (Figure 5). We further evaluated PA-induced apoptosis in RPTECs using Annexin V/SYTOX staining. PA significantly increased the proportion of apoptotic cells, and 5-ALA rescued lipoapoptosis. Finally, Zn-PpIX inhibited the protective effects of 5-ALA (Figure 6). These findings indicate that 5-ALA attenuated PA-induced cellular apoptosis through induction of HO-1. 

## 3. Discussion

In the present study, we showed PA-induced ER stress and apoptosis in renal tubular epithelial cells. Moreover, 5-ALA suppressed GRP78 expression, resulting in attenuated caspase-3/7 activity and cellular apoptosis. The protective effect of 5-ALA against lipotoxicity was mediated by HO-1 induction. Furthermore, 5-ALA significantly suppressed a HO-1 repressor, BACH1. These findings suggest that HO-1 induction is a potential therapeutic target for fatty acid-induced ER stress and kidney injury. 

Obesity causes the accumulation of lipids in the adipose tissue, which primarily serves as the site to store excess lipids in the form of triglycerides. This prevents the non-adipose tissue from excessive lipid accumulation and lipotoxicity. Exposure to fatty acids that exceeds oxidation capacity causes lipid droplet accumulation in hepatocytes, leading to NASH [24]. Steatosis due to excess nutrients or dysregulated lipid metabolism is also evident in the heart and kidneys. Cardiac steatosis and cardiomyopathy have been observed in mice fed a high-fat diet [25]. We previously reported that a high-fat diet induced renal tubular steatosis and renal dysfunction in C57BL/6 mice and fatty liver Shionogi-*ob/ob*, genetically modified mice exhibiting the NASH phenotype [13,14]. These associations between dysregulated lipid metabolism and multiple-organ steatosis suggest a common underlying pathophysiology of NASH, cardiomyopathy, and steatonephropathy. 

In this study, we used proximal tubular epithelial cells to investigate lipotoxicity-induced renal injury. Dysregulated energy metabolism in proximal tubular cells is a hallmark of renal injury [26]. We demonstrated PA-induced ER stress in renal tubular epithelial cells. PA is the major circulating long chain saturated fatty acid that causes cell injury by inducing various pathogenic stresses, including ER stress. PA-induced ER stress has been demonstrated in hepatocytes and cardiomyocytes [27,28]. In the kidney, ER stress is a significant contributor to acute and chronic renal injury of various etiologies [29,30]; thus, it could be a therapeutic target for steatonephropathy. We previously reported, in high-fat diet-fed mice, that ectopic lipid accumulation in the renal tubule causes ER stress and interstitial fibrosis. SGLT2 inhibitors improved the lipid metabolism and prevent steatosis in the kidney, leading to attenuated ER stress and interstitial fibrosis [13]. In addition to the excess fatty acid exposure, impaired lipid metabolism within the ER is another cause of ER stress. Because it is the primary location for the synthesis of phospholipids and sterols, the ER expresses a number of the enzymes and proteins that control lipid metabolism. Di-acyl glycerol transference 1, an enzyme dominantly expressed in the ER that catalyzes the triglyceride synthesis from fatty acids, is crucial for lipid metabolism and protects cells from excessive fatty acid exposure [31]. These observations indicate that ER stress is involved in organ injury under steatosis and is the promising target in dysregulated lipid metabolism. 

In the present study, we showed that *GRP78* expression was significantly increased by PA and significantly decreased after 5-ALA administration. GRP78 regulates the unfolded protein response in the ER to maintain homeostasis. In stressed cells, GRP78 induces several downstream transducers, such as activating transcription factor (ATF)-6, pancreatic ER kinase-like ER kinase (PERK), and inositol-requiring enzyme 1(IRE1α). These protect the cells by inducing ER chaperones to improve protein folding in the ER (ATF-6), activating antioxidant pathways (PERK), and accelerating the degradation of damaged proteins (IRE1α) [32]. If ER stress is severe or unresolved, the unfolded protein response becomes pro-apoptotic by inducing ATF-4 and C/EBP homologous protein (CHOP), the downstream mediator of PERK, leading to cellular apoptosis [22]. PERK, ATF-4, and CHOP play an important role in the progression of CKD under lipotoxicity. In a mouse model with a high-fat diet, improvement of lipid metabolism is associated with the suppression of renal tubular injury through GRP78 and CHOP pathway [13]. In the ER stress response cascade, cellular apoptosis is mediated by caspase-3. Caspase-3 is regulated directly by CHOP or indirectly by ATF-6 through B cell lymphoma-2 (bcl-2), which is an anti-apoptotic mediator playing a key role in the regulation of apoptosis in the unfolded protein response cascade. A previous report in human in vitro hepatocyte model showed that 5-ALA decreased PA-induced ATF-6 expression and increased bcl-2 expression, leading to suppression of caspase-3 activity [22]. We demonstrated that 5-ALA attenuates PA-induced ER stress, caspase activity, and cellular apoptosis, suggesting that 5-ALA is a potential therapeutic agent for lipotoxicity in renal tubules. 

We also found that 5-ALA induces *HMOX1* expression and reduces *BACH1* expression without affecting *NRF2* expression. HO-1, encoded by *HMOX1*, is an enzyme that catalyzes the degradation of heme to bilirubin, carbon monoxide, and iron. HO-1 expression dramatically increases under various stimuli, such as oxygen species and free radicals as a protective response against them. Importantly, heme is the most powerful stimulant of HO-1, and this is a reasonable reaction considering its heme degrading ability. Because HO-1 exhibits antioxidant and anti-inflammatory characteristics [32], it has a therapeutic potential to protect renal tubular cells from ER stress. Previous studies showed that HO-1 could prevent kidney injury by inducing antioxidant properties and suppressing proinflammatory cytokines [33]. HO-1 induction attenuated contrast-induced kidney injury through downregulation of inflammatory cytokines and inflammasome [34]. Similarly, HO-1 attenuated interstitial fibrosis and apoptosis in cyclosporine nephropathy [35]. Nrf2, a master transcriptional regulator of antioxidant proteins, positively regulates HO-1 and several studies have showed that Nrf2/HO-1 signaling is involved in the unfolded protein response pathway and prevents kidney injury [36]. A number of agents, including 5-ALA, have been suggested to upregulate Nrf2/HO-1 signaling [37]. However, the precise mechanisms of how 5-ALA upregulates Nrf2 or HO-1 remain to be clarified. Nrf2 is expressed in the cytoplasm in the basal condition, being bound to its repressor, the Kelch-like ECH-associated protein 1 (Keap1). This Nrf2-Keap1 complex undergoes ubiquitination and degradation and Nrf2 is kept at a low level. Oxidative modification of cysteine residues in Keap1 releases and activates Nrf2, allowing its entry to the nucleus. Nrf2 binds to an antioxidant response element of *Hmox1* for transcription and upregulates HO-1 expression [38]. In the present study, we observed remarkable *HMOX1* induction but no significant changes in *NRF2* expression after 5-ALA administration. Interestingly, we also observed that *BACH1* expression significantly decreased after 5-ALA administration in the renal tubular epithelial cells. Bach1 is the other transcriptional factor that directly regulates Hmox1 transcription under stress response [39]. 

**Figure 7:**
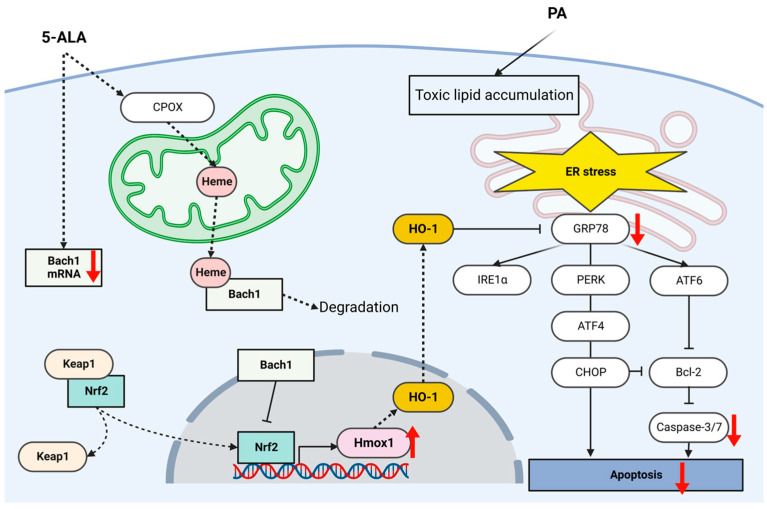
Scheme of the hypothesis of how 5-ALA attenuates lipoapoptosis. Exogenously administered 5-ALA decreases the Bach1 mRNA and is metabolized to heme. Heme binds to Bach1, inhibiting its expression and DNA-binding activity, which in turn engenders Nrf2 binding to the *Hmox1* promoter, leading to increased HO-1 expression. HO-1 downregulates GRP78 and subsequently attenuates ER stress-mediated pro-apoptotic signals. Suppression of caspase-3/7 activity attenuates apoptosis. 5-ALA, 5-aminolevulinic acid; CPOX, coproporphyrinogen III; PA, palmitic acid; HO-1, heme oxygenase-1; Bach1, BTB and CNC homology 1; Nrf2, nuclear factor erythroid 2-related factor 2; GRP78, glucose-regulated protein 78; ATF, activating transcription factor; PERK, endoplasmic reticulum kinase; IRE1α, inositol-requiring enzyme 1; CHOP, C/EBP homologous protein; Bcl-2, B cell lymphoma-2.

Bach1 represses the transcription of *Hmox1*. Bach1 has a counter-regulatory function against Nrf2 and is closely related to Nrf2 in the HO-1 regulatory system. Nrf2 and Bach1 form a heterodimer that directly regulates *Hmox1* transcription via interaction with its antioxidant response element of the promoter region [39]. Bach1 represses *Hmox1* transcription, interfering with Nrf2 by binding to its promoter region under normal conditions. Under excess heme conditions, heme binds to Bach1, leading to proteasomal degradation. Therefore, an increase in heme levels attenuates the Bach1-mediated repression of *Hmox1* transcription by decreasing Bach1 expression and interrupting its DNA-binding ability, enabling Nrf2 to bind to its antioxidant response element of the promoter region [40,41,42]. In this study, we observed the reduction in Bach1 mRNA levels after 5-ALA treatment. Therefore, we speculate that suppression of Bach1 resulted in upregulated *HMOX1* transcription, probably through accelerating Nrf2 binding to its promoter region. Increased expression in *SLC7A11*, a Nrf2-target gene, supports this hypothesis. Moreover, exogenously administered 5-ALA is absorbed into the cells and metabolized to coproporphyrinogen III, then transported into mitochondria. In the mitochondria, coproporphyrinogen III is further metabolized to heme [15]. It should be noted that free heme is toxic. Heme has high affinity towards biological membranes and stimulates reactive oxygen species and inflammatory cytokine generation, leading to the oxidative stress, mitochondrial DNA damage, and activation of the unfolded protein response pathways [32]. A previous study showed that heme increased the expressions of GRP78, PERK, IRE1α, and ATF6 in aortic smooth muscle cells, indicating that heme is a direct inducer of ER stress [43]. Additionally, they found that heme scavenger proteins could effectively attenuate the heme-induced ER stress. HO-1 is the essential enzyme to catabolize heme to biliverdin converted to bilirubin and carbon monoxide. Bilirubin has an antioxidant effect and carbon monoxide and carbon monoxide-releasing molecules have anti-inflammatory character. We speculate that although exogenous 5-ALA is potentially harmful due to heme generation, HO-1 induction counter-regulates to catabolized heme, resulting in the attenuated ER stress (Figure 7). 

There are several limitations in the present study. We evaluated the major genes involved in heme biosynthesis, such as *HMOX1*, *NRF2*, and *BACH1,* in this study. HO-1 can be induced by the other transcription factors, such as hypoxia inducible factor, which is not included in this study. Although the major gene and protein expressions were measured, these did not include whole molecules relevant to ER stress and heme biosynthesis pathway. The upregulation of Hmox1 transcription by Nrf2 is not only mediated by its entry into the nucleus but also by phosphorylation. Bach1 mRNA reduction suggested that mechanisms other than heme-mediated Bach1 degradation are also involved in the 5-ALA-mediated HO-1 induction, and this warrants further investigation. However, the finding that 5-ALA significantly decreased *BACH1* expression suggests that 5-ALA induces HO-1 expression through downregulating the transcription repressor of Hmox1. Because this study is performed using renal tubular cell lines, induction of HO-1 by 5-ALA and its effect has not yet been determined. Further investigations, including animal experiments, are required to elucidate its therapeutic potential. 

## 4. Materials and Methods

### 4.1. Cell Lines and Cell Culture

The RPTEC line was obtained from the American Type Culture Collection (Rockville, ML, USA). The cells were maintained in Dulbecco’s modified Eagle’s medium (DMEM)/F12 supplemented with 10% fetal bovine serum, 1% l-glutamine, and 1% antibiotic G418. The cells were cultured in a humidified incubator at 37 °C in an atmosphere of 95% air and 5% CO_2_. Palmitic acid (PA, P5585-10G; Sigma-Aldrich, St. Louis, MO, USA), 5-ALA (purity > 95%, AL-05-01; Cosmo Bio Co., Tokyo, Japan), and zinc protoporphyrin IX (Zn-PpIX, SC-200329; Cosmo Bio Co., Tokyo, Japan) were purchased for cell stimulation and treatment. PA was dissolved in isopropanol at 37 °C to a concentration of 100 mM, as previously described [21]. The final concentration of PA was adjusted to 10, 50, 100, and 200 μM in DMEM containing 1% BSA. BSA-bound PA was freshly prepared in each experiment. Then, 5-ALA was dissolved in serum-free DMEM and administered at a final concentration of 200 μM [22]. Zn-PpIX, an HO-1 inhibitor, was dissolved in dimethyl sulfoxide to a concentration of 1 mM and administered to the medium at a final concentration of 10 μM. A lipid droplet-specific fluorescent probe for live-cell imaging, Lipi-Green (LD-02, Dojindo Laboratories, Kumamoto, Japan), was used for lipid staining according to the manufacturer’s protocol. 

### 4.2. Gene Expression Analysis

Gene expression was analyzed by real-time RT-PCR as previously described [44]. Total RNA was extracted after a brief wash in phosphate-buffered saline from cells using a miRNeasy Mini Kit (QIAGEN, Tokyo, Japan) according to the manufacturer’s protocol. Extracted RNA was quantified using a BioSpec-nano spectrophotometer (Thermo Fisher Scientific, Tokyo, Japan). Reverse transcription was performed using a High-Capacity cDNA Reverse Transcription Kit (Thermo Fisher Scientific, Tokyo, Japan) with 2 μg of the extracted total RNA in a final volume of 20 μL containing 1× RT buffer, 4 mM dNTP mix, 1× RT random primer, 50 units of multiscribe reverse transcriptase, 20 units of RNase inhibitor, and nuclease-free water at 25 °C for 10 min, followed by 37 °C for 120 min and 85 °C for 5 min. Quantitative PCR analyses were performed to measure the mRNA levels of the target genes in 20 μL aliquots containing 1 μL cDNA, 4 μL LightCycler Fast Start DNA Master PLUS SYBER Green (Roche Diagnostics, Tokyo, Japan), 0.5 μM primer, and 14.6 μL nuclease-free water using the Real-Time PCR LightCycler 1.5 Complete System (Roche Diagnostics) at 95 °C for 10 min, followed by 45 cycles of 95 °C for 10 s, 60 °C for 10 s, and 72 °C for 10 s. Gene expression was calculated using the 2^−ΔΔC(T)^ method [45]. The cycle passing threshold was determined using the LightCycler Software version 5.5.28 (Roche Diagnostics, Tokyo, Japan). The forward and reverse primer sequences used for real-time RT-PCR are summarized in Table 1. ACTB, encoding β-actin, was used as the internal control for normalization. Quantification is based on five repetitive experiments. 

### 4.3. Protein Expression Analysis

The cells were lysed in RIPA buffer (Fujifilm Wako Pure Chemical, Tokyo, Japan) containing protease and phosphatase inhibitor (Roche Diagnostics). Samples were mixed with Laemmli buffer containing 2-mercaptoethanol. Thirty micrograms of protein were loaded for SDP-PAGE gel and transferred to nitrocellulose membranes. Western blotting was performed using established protocols [44]. Densitometry analysis was performed using ImageJ software 1.52a (National Institute of Health, Bethesda, MD, USA).

### 4.4. Quantification of Caspase Activity and Cellular Apoptosis

Caspase activity was measured using the Cell Even Caspase-3/7 Detection Reagent (Thermo Fischer Scientific, Waltham, MA, USA). Cell apoptosis was analyzed by staining with the Annexin V-FITC Apoptosis Detection Kit Plus (BioVision, Milpitas, CA, USA) and Hoechst 33342 (Thermo Fisher Scientific, Waltham, MA, USA) as previously described [22,46]. An all-in-one Fluorescence Microscope BZ-X800 (KEYENCE, Osaka, Japan) was used to acquire the fluorescence images. At least four fields at each condition were randomly captured for quantification.

### 4.5. Statistical Analysis

All values are expressed as means ± SEM. One-way analysis of variance with Tukey’s post hoc test was used to compare each group. Statistical significance was set at *p* < 0.05. GraphPad Prism 7.0 (GraphPad Software, San Diego, CA, USA) was used for statistical analysis.

## 5. Conclusions

In the present study, we demonstrated that 5-ALA prevented PA-induced ER stress and cellular apoptosis in renal tubular cells. The finding that 5-ALA induces HO-1 suggests that 5-ALA potentially improves ER stress response and redox pathways. Our results indicate that heme biosynthesis pathways including HO-1 are involved in the 5-ALA-mediated protective effect and HO-1 induction is a potential therapeutic target for fatty acid-induced ER stress and kidney injury.

## Figures and Tables

**Table 1:** List of primers for PCR analysis.

Gene Product	Accession ID	Forward Primer (5′ to 3′)	Reverse Primer (5′ to 3′)
*GRP78*	NM_005347.5	CGTGGAATGACCCGTCTGTG	CCAGCGTCTTTGGTTGC
*HMOX1*	NM_002133.3	GCCAGCAACAAAGTGCAAG	GAGTGTAAGGACCCATCGGA
*NRF2*	NM_006164.5	CAGCGACGGAAAGAGTATGA	TGGGCAACCTGGGAGTAG
*BACH1*	NM_206866.3	CTCAGCCTTAATGACCAGCGG	GCCTACGATTCTTGAGTGGAAG
*NQO1*	NM_001025434.2	GTGATATTCCAGAGTAAGAAGGCAG	ATTCTCCAGGCGTTTCTTCCAT
*SLC7A11*	XM_054349496.1	GGTGGTGTGTTTGCTGTC	GCTGGTAGAGGAGTGTGC
*ACTB*	NM_001101.5	CATGTACGTTGCTATCCAGGC	CTCCTTAATGTCACGCACGAT

## Data Availability

Not applicable.
